# Current Status of Nurse Team Resilience in China: A Latent Profile Analysis

**DOI:** 10.1155/jonm/2487832

**Published:** 2025-10-22

**Authors:** Zhiwei Wang, Jian Liu, Huimin Wei, Xueqing Song, Zeping Yan, Shicai Wu, Xiaorong Luan

**Affiliations:** ^1^School of Nursing and Rehabilitation, Cheeloo College of Medicine, University of Shandong, Jinan, Shandong, China; ^2^University of Health and Rehabilitation Sciences, Qingdao, Shandong, China; ^3^Department of Nursing, Shandong Provincial Hospital, Jinan, Shandong, China; ^4^Beijing Bo'ai Hospital, China Rehabilitation Research Center, Beijing, China; ^5^Department of Infection Control, Qilu Hospital of Shandong University, Jinan, Shandong, China

**Keywords:** latent profile analysis, nurses, team, team resilience

## Abstract

**Background:**

High job stress is the primary cause of nurse shortages, while research on resilience training for nurse teams is still rising. To inform the design of effective resilience interventions, understanding the training needs of nurse teams at the outset is essential.

**Objective:**

This study aimed to investigate the current status, patterns, and influencing factors of nurse team resilience.

**Methods:**

A multicenter cross-sectional survey was conducted among 217 Chinese nurse teams (comprising 1618 individual nurses) recruited through stratified convenience sampling. Latent profile analysis was used to identify nurse teams' resilience profiles based on standardized instruments. Multinomial logistic regression was employed to explore the demographic and team variables that were linked to the different profiles.

**Results:**

Latent profile analysis revealed the three-profile model had the best fit. The three profiles were named “low team resilience” (Class 1, n = 47, 21.659%), “high team resilience” (Class 2, n = 76, 35.023%), and “medium team resilience” (Class 3, *n* = 94, 43.318%). Gender, educational level, satisfaction with salary, and hospital level differed significantly across the three profiles in multivariate models. Nurse teams with no men (OR = 0.407, *p* < 0.05), with no members having postgraduate education (OR = 0.223, *p* < 0.05), with members who were dissatisfied with their salary (OR = 0.237, *p* < 0.01), or those from secondary hospitals (OR = 0.243, *p* < 0.01) were more likely to be assigned to the low team resilience profile.

**Conclusions:**

This study provides insights about team resilience that are useful for nurse managers and hospital administrators. In particular, when designing interventions and developing policies, managers can identify characteristics that need to be prioritized to achieve favorable outcomes.

## 1. Introduction

Nurse shortages are a major public health and social problem worldwide [1]. According to estimates by the International Council of Nurses, the current shortage is up to 13 million nurses globally; this problem is likely to worsen given the increasing healthcare needs in aging societies, particularly those in developing countries with limited access to healthcare (International Council of Nurses) [2]. In China, there are only 4.7 million registered nurses, representing 3.34 nurses for every 1000 citizens [3, 4], highlighting the seriousness of this public health problem. The extent of nursing human resources has a critical impact on patient outcomes. A Lancet survey of 420,000 patients and 26,000 nurses from nine European countries and 300 hospitals found that for each additional patient cared for by nurses, patient mortality increased by 7%, while such mortality decreased by 7% for each 10% increase in the number of nurses [5]. In addition, nurse shortages significantly increase the chance that nurses encounter issues at work, such as needlestick injuries, medication errors, and decreased productivity [6]. Therefore, understanding the reasons for and finding methods to cope with nurse shortages is an urgent task.

High job stress is the most important cause of nurse shortages [7]. Compared with general occupational groups, nurses work in uniquely complex environments, have nonstandard working hours, and have to face a variety of uncertainties and complex stress situations; serious feelings of professional withdrawal and fatigue are common [8]. Against this background, scholars are increasingly focusing on the way in which nurse resilience, especially at the level of nursing teams, can be a crucial method of reducing nurse shortages and enhancing their professional performance [9, 10]. Team resilience, also known as team toughness, refers to the strength that helps a team successfully cope with and recover from persistent stress or challenges [11–13]. As a focus of team stress research, team resilience can explain why, when faced with the same pressures, difficulties, and adversities, some nurse teams adapt while others do not. The conceptual foundation of this construct, primarily defined through its adaptive process and operationalized within frameworks like the input-mediator-output (IMOI) model, has been extensively developed and validated in Western countries; research has confirmed the significant positive effects of team resilience in the context of workplace stress [14–18]. Similarly, research on team resilience has been frequently conducted in China, with studies extending to encompass the nursing population [13, 19–21]. However, a critical examination of the current research landscape reveals several limitations.

First, the current status of nurse team resilience in China is not well understood. Existing studies have predominantly focused on demonstrating the positive outcomes of resilience, such as its association with reduced turnover intentions and improved team performance [13, 20]. While valuable, this outcome-oriented approach has overshadowed the foundational need to systematically assess and profile the baseline levels of resilience among nurse teams. There is a scarcity of research that captures the heterogeneous nature of team resilience, meaning we do not know what proportion of teams possess high, medium, or low resilience, or what patterns of strengths and weaknesses exist across different resilience capabilities. Second, and consequently, the existing research problems are not clearly articulated. Without a clear understanding of the heterogeneity in team resilience profiles, it is difficult to identify which specific teams are most vulnerable and in need of targeted interventions. Furthermore, the factors that predispose a team to a particular resilience profile remain largely unexplored. While demographic factors like individual gender and education are often studied, their influence, when aggregated at the team level (e.g., teams with/without male members, teams with/without postgraduates), on the overall team's resilience profile is not well reported [22, 23]. This gap prevents nurse managers from developing precise, evidence-based strategies to build resilience, such as optimizing team composition or addressing specific organizational drivers like salary satisfaction.

Therefore, to address these gaps, this study intends to (i) investigate the current status of nurses' team resilience and identify its potential subtypes via LPA and (ii) examine the differences in demographic and team variables across these subtypes to understand the influencing factors of profile membership. This will provide a nuanced evidence base for tailoring team resilience training programs and stabilizing the nursing workforce.

## 2. Materials and Methods

### 2.1. Study Design and Sample

This study adopted stratified convenience sampling to select practicing nurses from five secondary and five tertiary hospitals in Shandong Province, China. Sample data were obtained annually beginning in 2020 as part of our long-term Nurses' Health Research Program; the data used for the current study are part of this long-term program. The inclusion criteria were (a) practicing nurses who held the People's Republic of China nursing practice certificate and (b) currently working in clinical nursing in a hospital. Nurses who were not involved in bedside care were excluded.

In this study, a “nurse team” was operationally defined as a work team composed of multiple nurses working within the same department or ward to jointly provide patient care [24]. This is typically the most natural and coherent unit for studying team-level phenomena in clinical settings, as members share common leadership (e.g., the same head nurse), goals, routines, and work environments, which are prerequisites for the development of shared perceptions measured by our team resilience instrument.

### 2.2. Measures

#### 2.2.1. Demographic Information and Characteristics of Nurse Teams

Individual demographic data were collected, including gender, age, educational level, marital status, number of children, satisfaction with salary, professional title, position, fringe benefits (such as parental leave, insurance, and retirement pensions), and years working in the current ward. To obtain the demographic characteristics of nurse teams, we aggregated the demographic information of individual nurses at the team level. Team gender characteristics were categorized as teams with and without men. Team age comprised the average of team members' ages. Educational level was categorized as teams having members with postgraduate education or not. Satisfaction with salary was categorized as teams with and without dissatisfied staff. Marital status refers to the number of unmarried nurses on the team. Number of children is the average number of children among team members. Professional title, position, and fringe benefits refer to the number of nurses with intermediate titles and above, head nurses, and satisfied respondents, respectively. Working years measures the average number of team members' working years in the current ward. The approach of aggregating individual-level demographic data to form team-level compositional characteristics is well established in organizational and team research [25].

Team characteristics included the team size, team type (such as internal, surgery, obstetrics, and gynecology), team maturity (i.e., head nurse's working years in the current ward), and hospital level (secondary or tertiary hospital) [13, 25, 26].

#### 2.2.2. Team Resilience

To measure team resilience, participants were asked to rate the Chinese version of the Analyzing and Developing Adaptability and Performance in Teams to Enhance Resilience Scale (ADAPTER) [27]. The translation, cultural adaptation, and validation of the ADAPTER scale for use with Chinese nursing populations have been reported in our previous studies [25, 28], which demonstrated excellent reliability and validity. The ADAPTER covers seven dimensions, namely, responding (8 items), shared transformational leadership (13 items), learning (4 items), anticipating (5 items), monitoring (10 items), cooperation with other departments (8 items), and heedful interrelating (3 items). Each item was rated on a 5-point Likert scale from 1 (*strongly disagree*) to 5 (*strongly agree*). Total scores ranged from 51 to 255, with higher scores demonstrating greater team resilience. The scale was completed by all individual members of each nurse team, including both frontline staff nurses and the head nurse. In this study, Cronbach's alpha was calculated as 0.993 for the overall ADAPTER scale; individual dimensions ranged from 0.918 to 0.981.

### 2.3. Data Collection

Following methodological guidance for multilevel research [29], when performing a team-level study, the team-level sample size should be at least 51 and there should be at least 5 team members at the individual level (i.e., team size). To ensure adequate statistical power, we sampled 217 nurse teams with 5 or more nurses. In this study, the Questionnaire Star application (an online data collection platform) was used to design an anonymous online survey that was distributed through WeChat (Tencent Holdings Limited). After obtaining informed consent from the nursing department of each selected hospital, a unique link or QR code to the online questionnaire was sent to the nursing department who subsequently forwarded and promoted the link or QR code to the target population via a WeChat group. A consent form and description of the study's purpose were included at the start of the questionnaire. Participants were able to access the questionnaire after providing informed consent.

### 2.4. Statistical Analysis

In the subsequent analysis, we first aggregated individual scores to the team level, which was necessary and justified given that our study focused on team resilience (i.e., the shared perception of the attitudes and beliefs of the team members) within the defined ward/departmental units [13, 28]. To validate team-level data aggregation, inter-rater agreement was evaluated by calculating the r_wg_ (acceptable range > 0.7). Intraclass correlations and reliability of the group means were assessed by calculating the intraclass correlation coefficients (ICC 1; acceptable range > 0.05) and (ICC 2; acceptable range > 0.5) [30, 31].

Statistical analyses were performed using SPSS software (Version 25.0) and Mplus software (Version 8.3). For the descriptive statistics, continuous variables were displayed as mean (standard deviation [SD]) for variables with a normal distribution and as median (interquartile range [IQR]) for the others; categorical variables were expressed as frequency and percentage (%). To identify the latent profiles (subtypes) of team resilience among nurse teams, LPA was performed using the seven dimensions of team resilience.

LPA is a person-centered approach that identifies subtypes based on the heterogeneous characteristics of samples. The samples in each subtype share attributes, helping us to identify potentially diverse patterns of resilience in nurse teams [32]. The LPA model's goodness-of-fit was evaluated using the Akaike information criterion (AIC), Bayesian information criterion (BIC), sample-size-adjusted BIC (ABIC), Lo–Mendell–Rubin (LMR) adjusted likelihood ratio test, bootstrap likelihood ratio test (BLRT), and entropy index. AIC, BIC, and ABIC represent model fitness and parsimony [33, 34]. LMR and BLRT reflect the difference in model fit between the class *k* − 1 and *k* models, with lower *p* values indicating a better fit of the latter compared with the former [34]. Entropy reflects the accuracy of LPA model classification [34]. The criterion for a good fit is that the values of AIC, BIC, and ABIC should be lower, significant *p* values for LMR and BLRT should be lower, and the value of entropy should be higher (converging to 1). In addition, each subtype should comprise at least 5% of the total sample to avoid overstratification [35, 36].

One-way ANOVA and Chi-square (*χ*^2^) tests were used to identify differences in continuous and categorical variables, respectively, among the profiles. Post hoc analysis with a least significant difference (LSD) *t*-test was used to examine differences in every pair of profile comparisons. In addition, multinomial logistic regression analysis was performed using variables selected by the univariate analyses as independent variables and different profiles of team resilience as dependent variables.

### 2.5. Ethical Considerations

All procedures followed the Declaration of Helsinki, and the protocol was reviewed and approved by the Ethics Committee of Scientific Research of Shandong University School of Nursing and Rehabilitation (Ethics Approval No. 2021-R-131). Moreover, responses were kept strictly confidential and anonymous.

## 3. Results

In total, responses from 217 nurse teams comprising 1618 nurses were received. Detailed demographic characteristics of the 217 nurse teams are shown in Table 1. Each team included 5–13 nurses (mean = 7.46, SD = 1.85). Of these, 25.81% were surgery teams, 57.14% were from secondary hospitals, and the average team maturity was 22.44 years (SD = 19.47). Furthermore, the r_wg_ values for team resilience were > 0.8. Intraclass correlation coefficient (1) ranged from 0.08 to 0.1 and (2) was > 0.5, indicating appropriate team-level aggregation of data (see Supporting Table S1).

### 3.1. Latent Profile Models

The LPA results for team resilience are shown in Table 2. The three-class model showed the best fit. It had the lowest AIC (4595.084), BIC (4696.481), and ABIC (4601.415). Meanwhile, the LMR test (*p* = 0.0368) and BLRT (*p* < 0.001) indicated that the three-class model was statistically significant at *α* = 0.05. This model also had a higher entropy value (0.966), and the proportion of nurse teams in the smallest subtype was 21.7% (larger than the 5% threshold). Therefore, this study used the three-profile model for subsequent interpretation and analysis.

**Figure 1:**
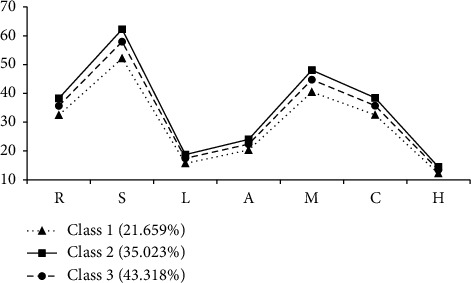
Three profiles of team resilience. Note: R, responding; S, shared transformational leadership; L, learning; A, anticipating; M, monitoring; C, cooperation with other departments; H, heedful interrelating.

Table 3 and Figure 1 show the distribution of team resilience in the three-profile model. The mean total and dimensional scores of the ADAPTER significantly differed among the three classes (all *p* < 0.001). Class 1, the smallest subtype (*n* = 47, 21.659%), had the lowest levels of team resilience. Class 2 formed the second-largest subtype (*n* = 76, 35.023%) and was characterized by the highest levels of team resilience. Class 3 was the largest subtype (*n* = 94, 43.318%) and had medium levels of team resilience. As Figure 1 shows, although the profiles differed in overall resilience scores, their response patterns were similar (e.g., the score levels for learning, anticipating, and heedful interrelating dimensions were much lower than the other dimensions). Based on the results, Class 1 was named the “low team resilience” subtype, Class 2 was labeled as the “high team resilience” subtype, and Class 3 was labeled as the “medium team resilience” subtype.

### 3.2. Characteristic Variations Among Profiles

In terms of demographic and team differences among the three profiles, ANOVA and *χ*^2^ tests implied significant differences across profiles in gender, educational level, satisfaction with salary, team maturity, and hospital level (all *p* < 0.05; Table 4). In particular, Class 1 had the lowest proportion of teams with no men, with no postgraduates, with no members who were dissatisfied with their salaries, and from secondary hospitals; they had the second-largest levels of team maturity.

### 3.3. Factors Associated With Team Resilience Profiles

The multinomial logistic regression analysis results are shown in Table 5. In this study, the three profiles (Classes 1–3) were used as the outcome variable, and the independent variables were gender, educational level, satisfaction with salary, team maturity, and hospital level. Class 1 (“low team resilience” subtype) was set as the base outcome (reference group). The final results indicated that gender, educational level, satisfaction with salary, and hospital level were linked to different nurse team resilience profiles, while team formation years had no significant impact. In the comparison between the low and medium team resilience subtypes (Classes 1 and 3), teams with no men were more likely to belong to Class 1 compared with teams with men (OR = 0.407, *p* < 0.05). Secondly, comparing Classes 1 and 2 (low vs. high), nurse teams with no postgraduates were more likely to be grouped in Class 1 (OR = 0.223, *p* < 0.05). In terms of satisfaction with salary and hospital level, teams with dissatisfied staff and those from secondary hospitals were more likely to fall into Class 1 (OR = 0.237, *p* < 0.01; OR = 0.243, *p* < 0.01). In summary, the low team resilience subtype was characterized by nurse teams with no men, with no postgraduates, with dissatisfied staff, and teams from secondary hospitals.

## 4. Discussion

This study identified three different profiles of nurse team resilience: low (Class 1; *n* = 47, 21.659%), high (Class 2; *n* = 76, 35.023%), and medium (Class 3; *n* = 94, 43.318%). The resilience of the largest proportion of nurse teams was at a moderate level. Son and Ham [10] also identified a moderate level of team resilience in their study on nurse teams in Korea. Gender, educational level, satisfaction with salary, and hospital level were determined as influencing factors of nurse team resilience. Nurse teams with no men, with no postgraduates, with dissatisfied staff, and from secondary hospitals were more likely to be assigned to the low team resilience subtype (Class 1). Notably, compared with other capabilities in the ADAPTER scale, nurse teams (regardless of overall level) demonstrated lower scores for “learning,” “anticipating,” and “heedful interrelating.” This finding may be explained as follows.

Learning is defined as the capability of team members to learn from their experiences, which can be specifically divided into explorative and exploitative learning (i.e., team creativity and team task completion) [27, 37, 38]. Explorative learning refers to exploring new possibilities in a creative way, such as developing new nursing practices to prevent adverse patient events. Exploitative learning emphasizes the execution of an established task plan in a skillful and efficient way, and is closely related to the completion of tasks according to standardized procedures [39, 40]. In China, whether novice or senior, nurses are subjected to a series of training programs by their organization, which are conducted only with a view to ensuring nurses are able to perform their jobs quickly according to standardized procedures; creativity in nurse teams is often left undeveloped [41]. In addition, most nurses do not conduct systematic and standardized recertification after qualifying for training [42]. Thus, their capability for explorative learning may not have been developed.

As for anticipating and heedful interrelating capabilities, these refer to the ability of the team to anticipate the future development of events and achieve effective cooperation among team members when faced with unexpected situations (e.g., nurse–patient conflicts and adverse events), respectively [13, 27, 37]. The low level of capability in these areas may be explained as follows. First, this study was conducted in Shandong province, China (known as the Confucian cradle). This region has always emphasized the value of benevolence, and both patients and caregivers are typically empathetic and understanding, so there are few nurse–patient conflicts; thus, they may have had few opportunities to develop relevant skills for dealing with these situations [43, 44]. Second, with the adjustment of China's nursing program as a state-controlled distribution program [45], nurses must have at least a bachelor's degree in order to gain employment in high-level hospitals. This suggests that nurses working in high-level hospitals typically possess a high level of professionalism. Notably, the sample of our research was selected from secondary and tertiary hospitals, wherein the majority of the research team consisted of nurses with postgraduate degrees. Hence, relative to other samples, these nurse teams may rarely encounter adverse events. Given these findings, strengthening the learning, anticipating, and heedful interrelating capabilities of nurse teams should be a specific focus of programs designed to improve team resilience.

Among the demographic and team characteristics, gender, educational level, satisfaction with salary, and hospital level differed across the potential profiles. Nurse teams with no men, with no postgraduates, with dissatisfied staff, and teams from secondary hospitals had more probability of being assigned to the low team resilience class than those with men, with postgraduates, with no dissatisfied staff, and those from tertiary hospitals. We suggest the following explanations for these results. First, the addition of male nurses may bring diverse skills and perspectives to the care team, thereby enriching the team's overall capabilities [46, 47]. For example, they may demonstrate different strengths in physical handling, emotional management, or crisis response. In addition, male nurses are often physically stronger, which may prove useful in sharing the burden of physical labor under heavy workloads, especially when dealing with emergencies or critically ill patients (e.g., emergency, critical care, and other units) [48]. This physical support may reduce stress on other team members. Furthermore, the addition of male nurses may break down traditional gender role restrictions, thus promoting cooperation and understanding among team members and reducing stress and conflict. In summary, teams with male nurses may be more diverse, which not only promotes teamwork and mutual psychological support, but also improves the team's overall resilience.

As for education level, nurse teams with postgraduates are likely to demonstrate stronger critical thinking and have deeper medical and nursing knowledge [49]. In China, there is a fundamental difference between nurses' education at the bachelor and postgraduate stages. Although a bachelor's education equips nurses with a rich knowledge base and understanding of clinical practice, education at the postgraduate stage adds more training in critical thinking, problem solving, and evidence-based nursing practice [50]. Therefore, nurse teams with postgraduates may be better able to find solutions to stressful situations and respond more effectively to complex clinical situations, thereby reducing work stress. In addition, in China, where the level of nurses' education is directly proportional to promotion opportunities, nurse teams with postgraduates are likely to have improved career development prospects [51], which may increase their work motivation and resilience.

Regarding satisfaction with salary, nurse teams with dissatisfied members were found to be less resilient, which is consistent with previous evidence [52, 53]. Monthly income directly affects nurses' job satisfaction and productivity, and if salary levels do not meet nurses' expectations, they may feel a lack of adequate professional support and recognition, leading to burnout, emotional exhaustion, dehumanization, and low achievement [53]. These symptoms may mean they have inadequate support systems and coping resources in the face of stress, thus weakening their ability to cope with work challenges. As for hospital level, secondary hospitals in China generally have lower compensation levels and incentives and relatively fewer opportunities for career development [54]. This may explain why nurse teams at these hospitals were more likely to fall into the low team resilience profile.

### 4.1. Clinical Implications for Nursing Managements

With a growing number of hospitals using team structures, managers have begun to examine the strengths and powers of teams more closely, especially in the nursing scenario. The results of this study have important implications for nurse managers and policy makers in designing and implementing training programs to optimize the structure of nurse teams and improve their overall resilience and performance. The findings show that gender, education level, satisfaction with salary, and hospital level are strongly associated with nurses' team resilience profiles. According to the characteristics of the low team resilience profile, hospitals and nursing managers should consider increasing the diversity of nurse teams (especially the inclusion of male nurses) and promoting in-service education focusing particularly on critical thinking and problem-solving. Furthermore, incentives such as salary rewards should be used to motivate and increase the productivity of nurse teams, especially those from secondary hospitals.

When tailoring resilience training programs or designing incentives for nurse teams, managers can appropriately match these characteristics to develop optimal support programs or improve existing programs. Nurse managers may also consider investing in improving the capabilities of nurse teams in terms of learning, anticipating, and heedful interrelating, which were the primary weak points of team resilience in this study. For example, nurse managers could organize regular workshops or team meetings focused on explorative learning to facilitate nurses' communication and reflection on “what new interventions could be better than usual care,” strengthening positive work practices. In addition, managers could also organize regular live simulation trainings and debriefings featuring the management of different typical cases to help nurses understand best practices and strengthen their ability to adapt to unexpected situations. This would help develop optimal coping strategies and promote the development of resilient behaviors such as anticipating and cooperating in response to future adverse events.

### 4.2. Limitations

Despite its contributions, there are some limitations to this study. First, the geographical source of the sample was limited to one province in China, which may affect the generalizability of the findings. Studies with samples from multiple regions are necessary to enhance representativeness. Second, the sample size of 217 teams, while substantial for a team-level study and sufficient for identifying a well-fitting model in this analysis, may be considered moderate in the context of some simulation studies of LPA. Although our model fit indices (e.g., high entropy and significant LMR/BLRT) strongly support the chosen three-profile solution, future research with larger multicenter samples could further validate the stability and generalizability of these profiles and explore the potential for more nuanced subgroupings. Third, the reliance on the ADAPTER scale, while providing a validated multidimensional framework, may itself impose a structure that influenced the observed results. The scale's specific dimensions potentially prioritize certain aspects of resilience (e.g., responding and monitoring) over other unexplored but critical capabilities, such as a team's capacity for rapid innovation or informal resource integration. This conceptual boundary might have constrained the emergence of profiles with truly divergent patterns of strengths and weaknesses, potentially contributing to the identification of profiles that differ primarily in overall magnitude rather than in their fundamental architectural pattern. Future studies could benefit from employing mixed-methods or complementary tools to capture a more holistic picture of team resilience. Fourth, the factors considered to distinguish the three profiles were only based on demographic and team characteristic variables; future research should consider incorporating other relevant factors (e.g., team processes and organizational culture) to more precisely identify subtypes of nurse team resilience. Finally, LPA groupings are assigned based on the highest likelihood of a participant belonging to a particular latent profile; participants may not actually belong to a single group, so the results need to be interpreted with caution.

## 5. Conclusion

Based on LPA, this study explored the subgroup characteristics and factors influencing nurse team resilience at the team level. Within the seven dimensions of ADAPTER, we found three different profiles of team resilience, including “low team resilience” (Class 1, 21.659%), “medium team resilience” (Class 3, 43.318%), and “high team resilience” (Class 2, 35.023%). A similar pattern of change across the different dimensions of the ADAPTER was observed for the three classes, i.e., nurse teams performed poorly in terms of learning, anticipating, and heedful interrelating. In addition, we also found that gender, education level, satisfaction with salary, and hospital level were factors that influenced nurse teams' profile membership likelihood. In conclusion, this study emphasizes the need for nurse managers to recognize the importance of targeted training programs that incorporate nurses' specific forms of team resilience. Nurse managers and hospital administrators can tailor resilience training programs and improve the stability of the nursing workforce based on subgroup characteristics of nurse team resilience.

## Figures and Tables

**Table 1:** Demographic information and characteristics of nurse teams (*N* = 217).

Characteristics	Number (%)/mean ± SD	Characteristics	Number (%)/mean ± SD
Gender		Years working in the current ward	7.21 ± 3.15
Teams without men	54 (24.88)	Team size	7.46 ± 1.85
Teams with men	163 (75.12)	Team type	
Age	32.11 ± 2.92	Internal	45 (20.74)
Educational level		Surgery	56 (25.81)
Team without postgraduate	25 (11.52)	Obstetrics and gynecology	32 (14.75)
Team with postgraduate	192 (88.48)	Pediatrics	25 (11.52)
Marital status	1.88 ± 1.60	Emergency	24 (11.06)
No. of children	1.06 ± 0.43	Operating room	3 (1.38)
Satisfaction with salary		Critical care	32 (14.75)
Team with dissatisfied staff	111 (51.15)	Team maturity	22.44 ± 19.47
Team without dissatisfied staff	106 (48.85)	Hospital level	
Professional title	3 ± 1.71	Secondary hospital	124 (57.14)
Position	1.17 ± 0.40	Tertiary hospital	93 (42.86)
Fringe benefits	1.63 ± 1.27		

*Note:* Age, average age of team; Marital status, number of unmarried nurses in team; No. of children, average number of children in team; Professional title, number of nurses with intermediate titles and above in team; Position, number of head nurses in team; Fringe benefits, number of satisfied respondents in team; Years working in the current ward, average working years of team.

**Table 2:** Fit indices for various latent profile analysis models of team resilience (*N* = 217).

Model	AIC	BIC	ABIC	LMR (*p*)	BLRT (*p*)	Entropy	Proportion
1-Profile	6480.423	6527.742	6483.377	—	—	—	217
2-Profile	5314.451	5388.809	5319.094	0.079	< 0.001	0.944	77 (0.355)/140 (0.645)
3-Profile	4595.084	4696.481	4601.415	0.037	< 0.001	0.966	47 (0.217)/76 (0.350)/94 (0.433)
4-Profile	4213.584	4342.020	4221.603	0.218	< 0.001	0.969	9 (0.041)/48 (0.221)/89 (0.410)/71 (0.327)

*Note:* ABIC, sample-size-adjusted Bayesian information criterion; LMR, Lo–Mendell–Rubin adjusted likelihood ratio test.

Abbreviations: AIC, Akaike information criterion; BIC, Bayesian information criterion; BLRT, bootstrap likelihood ratio test.

**Table 3:** Scores on the seven dimensions of team resilience across three profiles (*N* = 217), mean (SD).

	Dimensions	Total sample (*n* = 217)	Class 1 (*n* = 47)	Class 2 (*n* = 76)	Class 3 (*n* = 94)	*F*	*p*
R	Responding	35.91 (2.49)	32.49 (1.61)^c^	38.31 (1.04)^a^	35.67 (1.27)^b^	303.14	< 0.001
S	Shared transformational leadership	58.20 (4.11)	52.17 (2.36)^c^	62.25 (1.81)^a^	57.94 (1.43)^b^	337.02	< 0.001
L	Learning	17.51 (1.34)	15.68 (0.77)^c^	18.74 (0.75)^a^	17.44 (0.69)^b^	255.38	< 0.001
A	Anticipating	22.51 (1.52)	20.33 (1.00)^c^	24.02 (0.56)^a^	22.37 (0.57)^b^	348.32	< 0.001
M	Monitoring	44.98 (3.06)	40.48 (1.76)^c^	48.06 (1.10)^a^	44.73 (1.10)^b^	414.62	< 0.001
C	Cooperation with other departments	36.00 (2.43)	32.53 (1.50)^c^	38.47 (0.87)^a^	35.74 (0.90)^b^	390.75	< 0.001
H	Heedful interrelating	13.53 (0.91)	12.21 (0.55)^c^	14.44 (0.34)^a^	13.45 (0.36)^b^	368.15	< 0.001
Total		228.64 (15.41)	205.90 (8.55)^c^	244.29 (5.63)^a^	227.35 (5.16)^b^	434.51	< 0.001

*Note:* Class 1, “low team resilience” subtype; Class 3, “medium team resilience” subtype; Class 2, “high team resilience” subtype.

^a^Highest scoring group in the post hoc analysis (*p* < 0.001).

^b^Medium scoring group in the post hoc analysis (*p* < 0.001).

^c^Lowest scoring group in the post hoc analysis (*p* < 0.001).

**Table 4:** Characteristic differences of team resilience across three profiles (*N* = 217).

Characteristics	Class 1 (*n* = 47)	Class 2 (*n* = 76)	Class 3 (*n* = 94)	*F*/*χ*^2^	*p*
Gender				7.77	0.021^∗^
Teams without men	19 (40.43%)	16 (21.05%)	19 (20.21%)		
Teams with men	28 (59.57%)	60 (78.95%)	75 (79.79%)		
Age	32.28 ± 3.19	31.87 ± 2.73	32.21 ± 2.94	0.40	0.674
Educational level				8.81	0.012^∗^
Team without postgraduate	10 (21.28%)	3 (3.95%)	12 (12.77%)		
Team with postgraduate	37 (78.72%)	73 (96.05%)	82 (87.23%)		
Marital status	2.13 ± 1.94	1.71 ± 1.47	1.90 ± 1.52	1.00	0.371
No. of children	0.97 ± 0.44	1.10 ± 0.40	1.07 ± 0.44	1.34	0.264
Satisfaction with salary				20.48	< 0.001^∗∗∗^
Team with dissatisfied staff	30 (63.83%)	23 (30.26%)	58 (61.70%)		
Team without dissatisfied staff	17 (36.17%)	53 (69.74%)	36 (38.30%)		
Professional title	3.06 ± 1.92	2.89 ± 1.55	3.04 ± 1.74	0.20	0.815
Position	1.26 ± 0.53	1.12 ± 0.33	1.16 ± 0.37	1.76	0.174
Fringe benefits	1.62 ± 1.23	1.41 ± 1.02	1.82 ± 1.44	2.23	0.110
Years working in the current ward	7.24 ± 3.44	6.69 ± 2.57	7.62 ± 3.38	2.11	0.126
Team size	7.60 ± 2.09	7.21 ± 1.72	7.59 ± 1.83	1.03	0.359
Team type				16.60	0.165
Internal	10 (21.28%)	13 (17.11%)	22 (23.40%)		
Surgery	8 (17.02%)	29 (38.16%)	19 (20.21%)		
Obstetrics and gynecology	5 (10.64%)	11 (14.47%)	16 (17.02%)		
Pediatrics	5 (10.64%)	8 (10.53%)	12 (12.77%)		
Emergency	8 (17.02%)	5 (6.58%)	11 (11.70%)		
Operating room	2 (4.26%)	0 (0.00%)	1 (1.06%)		
Critical care	9 (19.15%)	10 (13.16%)	13 (13.83%)		
Team maturity	20.36 ± 18.14	18.79 ± 15.83	26.44 ± 22.06	3.68	0.027^∗^
Hospital level				18.66	< 0.001^∗∗∗^
Tertiary hospital	35 (74.47%)	29 (38.16%)	60 (63.83%)		
Secondary hospital	12 (25.53%)	47 (61.84%)	34 (36.17%)		

*Note:* Age, average age of team; Marital status, number of unmarried nurses in team; No. of children, average number of children in team; Professional title, number of nurses with intermediate titles and above in team; Position, number of head nurses in team; Fringe benefits, number of satisfied respondents in team; Years working in the current ward, average working years of team.

^∗^ *p* < 0.05.

^∗∗∗^ *p* < 0.001.

**Table 5:** Multinomial logistic regression across three profiles of team resilience (*N* = 217).

Variables	“High team resilience” subtype		“Medium team resilience” subtype	
	*β*	OR (95% CI)	*β*	OR (95% CI)
Gender (ref: teams with men)				
Teams without men	−0.519	0.595 (0.246, 1.438)	−0.899^∗^	0.407 (0.185, 0.897)
Educational level (ref: team with postgraduate)				
Team without postgraduate	−1.500^∗^	0.223 (0.053, 0.947)	−0.374	0.688 (0.258, 1.837)
Satisfaction with salary (ref: team without dissatisfied staff)				
Team with dissatisfied staff	−1.439^∗∗^	0.237 (0.104, 0.542)	−0.029	0.971 (0.458, 2.062)
Team maturity	0.000	1.000 (0.977, 1.023)	0.016	1.016 (0.996, 1.037)
Hospital level (ref: tertiary hospital)				
Secondary hospital	−1.414^∗∗^	0.243 (0.103, 0.575)	−0.497	0.608 (0.269, 1.375)

*Note:* Reference subtype: “low team resilience” subtype (Class 1); “high team resilience” subtype, Class 2; “medium team resilience” subtype, Class 3.

Abbreviations: CI, confidence interval; OR, odds ratio.

^∗^ *p* < 0.05.

^∗∗^ *p* < 0.01.

## Data Availability

The datasets generated and/or analyzed during the current study are not publicly available due to privacy or ethical restrictions but are available from the corresponding author upon reasonable request.

## Supporting Information

Supporting Table 1. ICC (1), ICC (2), and r_wg_ values for each dimension of team resilience.
