# Supplementary material for: Current Status of Nurse Team Resilience in China: A Latent Profile Analysis
Source: J Nurs Manag. 2025 Oct 22;2025:2487832. doi: 10.1155/jonm/2487832 (PMC12571533; doi:10.1155/jonm/2487832)
Supplement: Supporting Information — Additional supporting information can be found online in the Supporting Information section. [file 2487832.f1.docx]

**Supplementary Table 1** ICC (1), ICC (2), and r_wg_ values for each dimension of team resilience

| Dimensions | r_wg_ | ICC (1) | ICC (2) |
| --- | --- | --- | --- |
| Responding | 0.94±0.15 | 0.08 | 0.53 |
| Shared transformational leadership | 0.96±0.13 | 0.10 | 0.57 |
| Learning | 0.88±0.18 | 0.08 | 0.53 |
| Anticipating | 0.93±0.13 | 0.10 | 0.57 |
| Monitoring | 0.96±0.12 | 0.10 | 0.57 |
| Cooperation with other departments | 0.95±0.12 | 0.10 | 0.58 |
| Heedful interrelating | 0.91±0.14 | 0.09 | 0.55 |

*Note*. r_wg_, inter-rater agreement; ICC (1) and ICC (2), intraclass correlation coefficients.
